# Supplementary figures and images for: MyD88-dependent influx of monocytes and neutrophils impairs lymph node B cell responses to chikungunya virus infection via Irf5, Nos2 and Nox2
Source: PLoS Pathog. 2020 Jan 30;16(1):e1008292. doi: 10.1371/journal.ppat.1008292 (PMC7012455; doi:10.1371/journal.ppat.1008292)

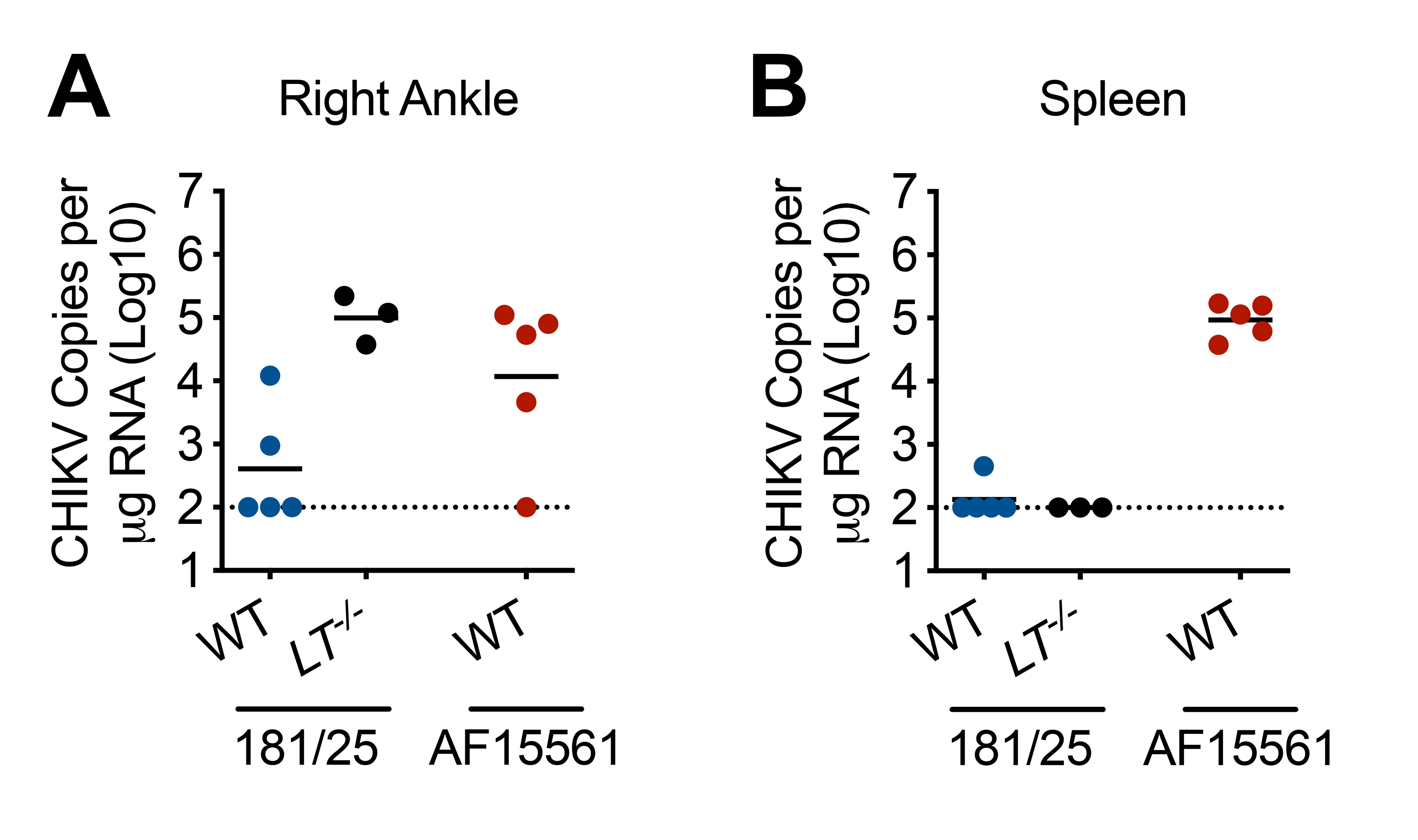

Supplement: S1 Fig — WT or congenic lymphotoxin alpha-deficient (LT-/-) C57BL/6 mice were inoculated with 103 PFU of CHIKV 181/25 or AF15561 in the left footpad. At 28 dpi, RNA was extracted from the (A) right ankle and (B) spleen and viral loads were determined by RT-qPCR. Data are from one independent experiment. (TIF) [file ppat.1008292.s001.tif]

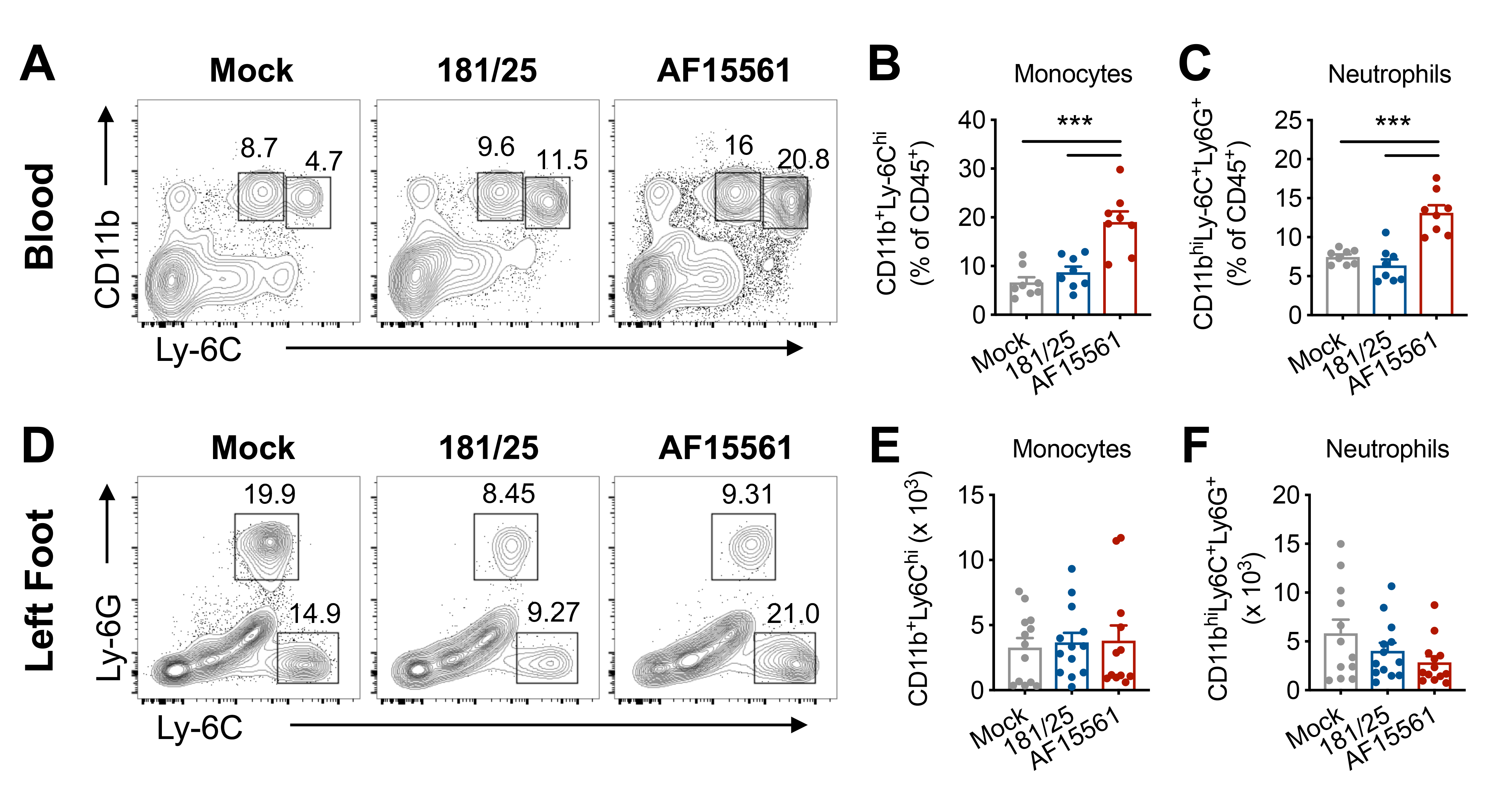

Supplement: S2 Fig — C57BL/6 mice were inoculated with PBS (mock) or with 103 PFU of CHIKV 181/25 or AF15561 in the left footpad. At 24 hpi, the blood and left foot were analyzed by flow cytometry. (A) Representative flow cytometry plots and percentages of (B) CD11b+Ly6Chi monocytes or (C) CD11bhiLy6C+Ly6G+ neutrophils in the blood. (D) Representative flow cytometry plots and numbers of (E) monocytes or (F) neutrophils in the left foot. Data are combined from two (A-C) or five (D-F) independent experiments (n = 4–21 per group). Statistical significance was determined by one-way ANOVA with Tukey’s post-test (***, P < 0.001). (TIF) [file ppat.1008292.s002.tif]

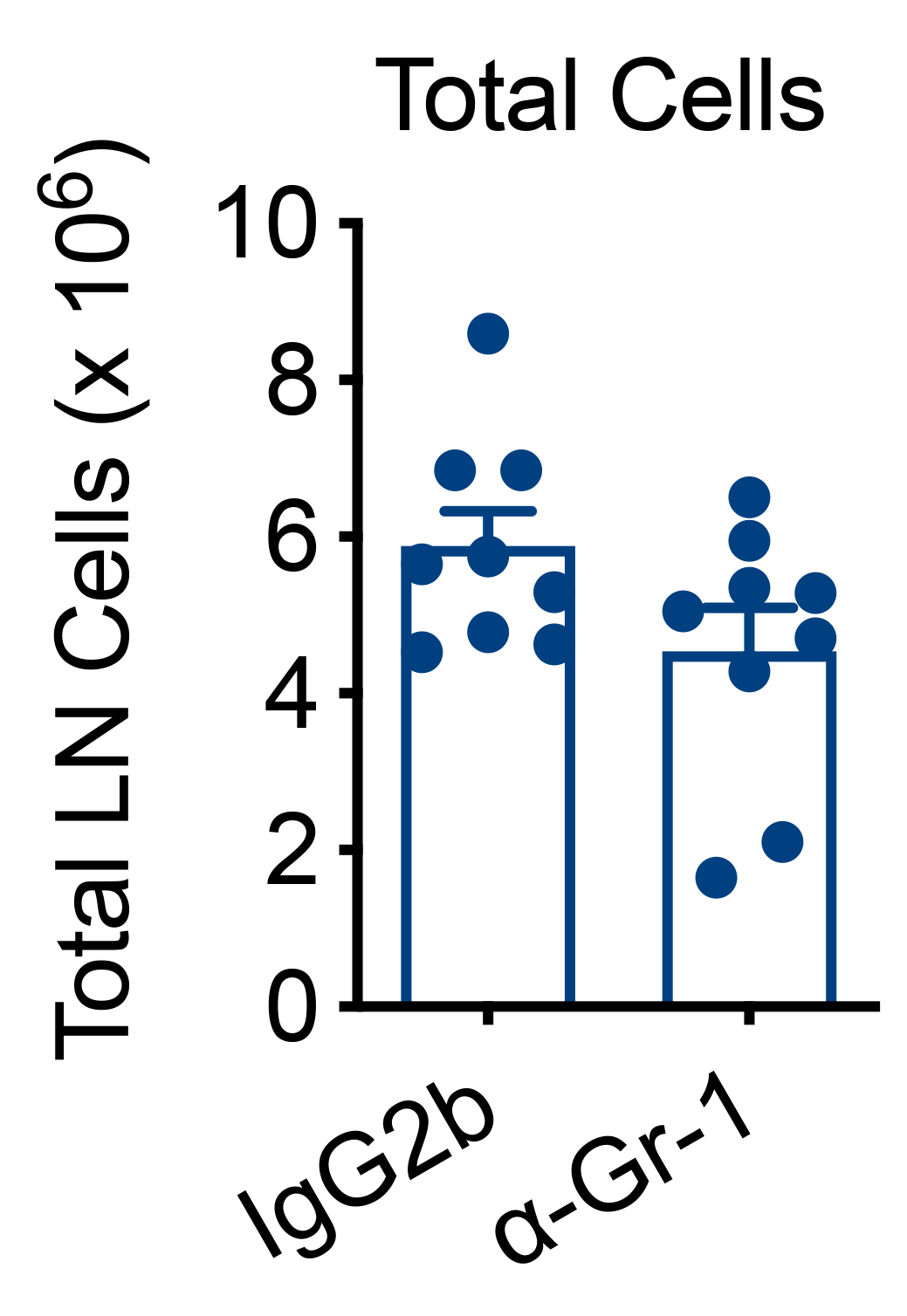

Supplement: S3 Fig — C57BL/6 mice were treated with 500 μg of IgG2b isotype control mAb or anti-Gr-1 mAb via intraperitoneal injection one day prior to inoculation with 103 PFU of CHIKV 181/25 in the left footpad. At 5 dpi, total cells in the dLN were enumerated. Data are combined from 2 independent experiments. (TIF) [file ppat.1008292.s003.tif]

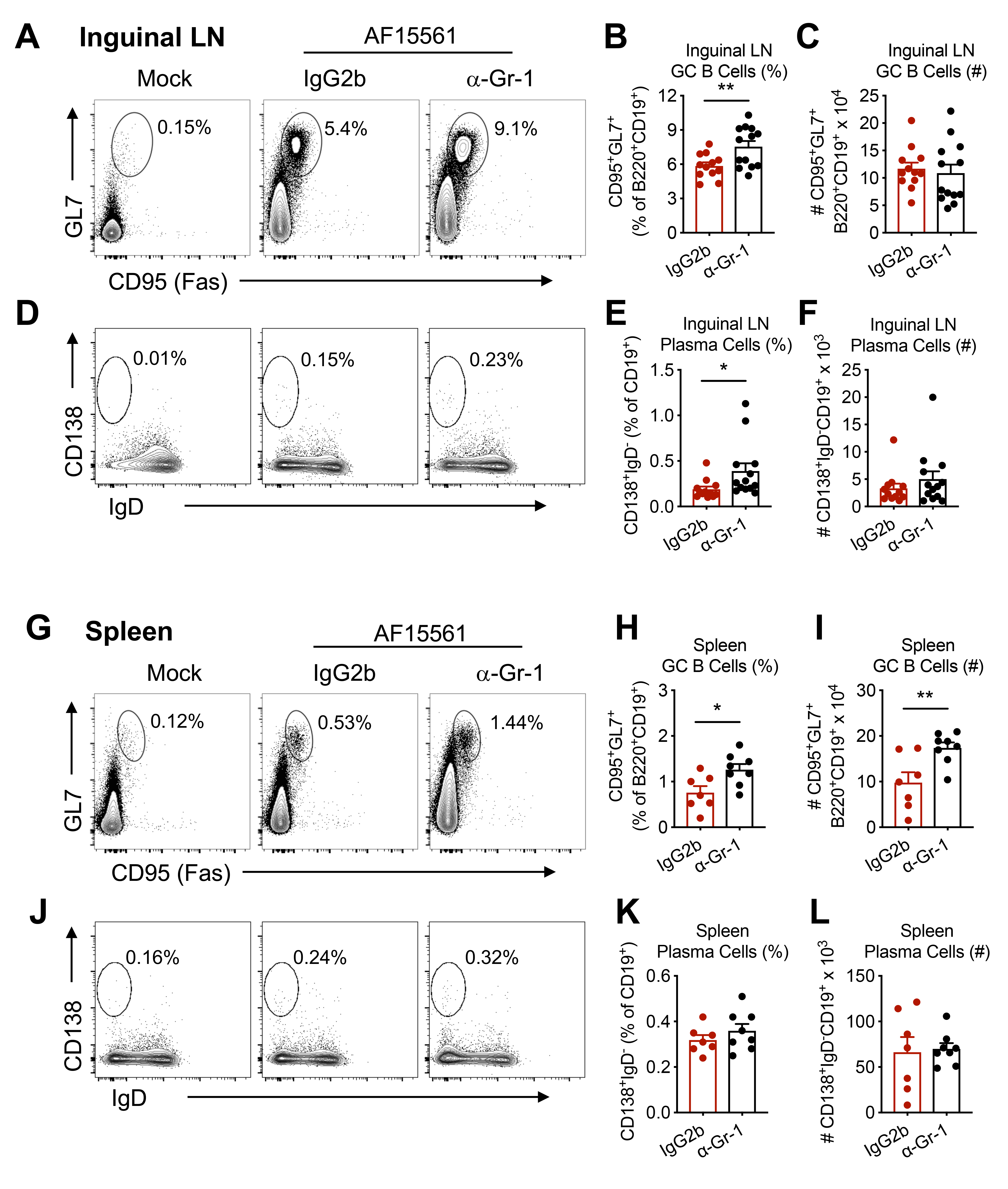

Supplement: S4 Fig — C57BL/6 mice were treated with 300–500 μg IgG2b isotype control mAb or anti-Gr-1 mAb i.p. one day prior to inoculation with 103 PFU of CHIKV AF15561 in the left footpad. (A) Representative flow cytometry plots of GL7+CD95+ GC B cells (gated on CD19+B220+ cells), (B) percentage and (C) total number of GC B cells in the left inguinal LN at 14 dpi. (D) Representative flow cytometry plots of CD138+IgD- plasma cells (gated on CD19+), (E) percentage and (F) total number of plasma cells in the left inguinal LN at 14 dpi. (G) Representative flow cytometry plots of GL7+CD95+ GC B cells (gated on CD19+B220+ cells), (H) percentage and (I) total number of GC B cells in the spleen at 14 dpi. (J) Representative flow cytometry plots of CD138+IgD- plasma cells (gated on CD19+), (K) percentage and (L) total number of plasma cells in the spleen at 14 dpi. Errors bars represent mean ± SEM. Data are derived from 2 independent experiments. Statistical significance was determined by Student’s t-test. *P < 0.05, **P < 0.01. (TIF) [file ppat.1008292.s004.tif]

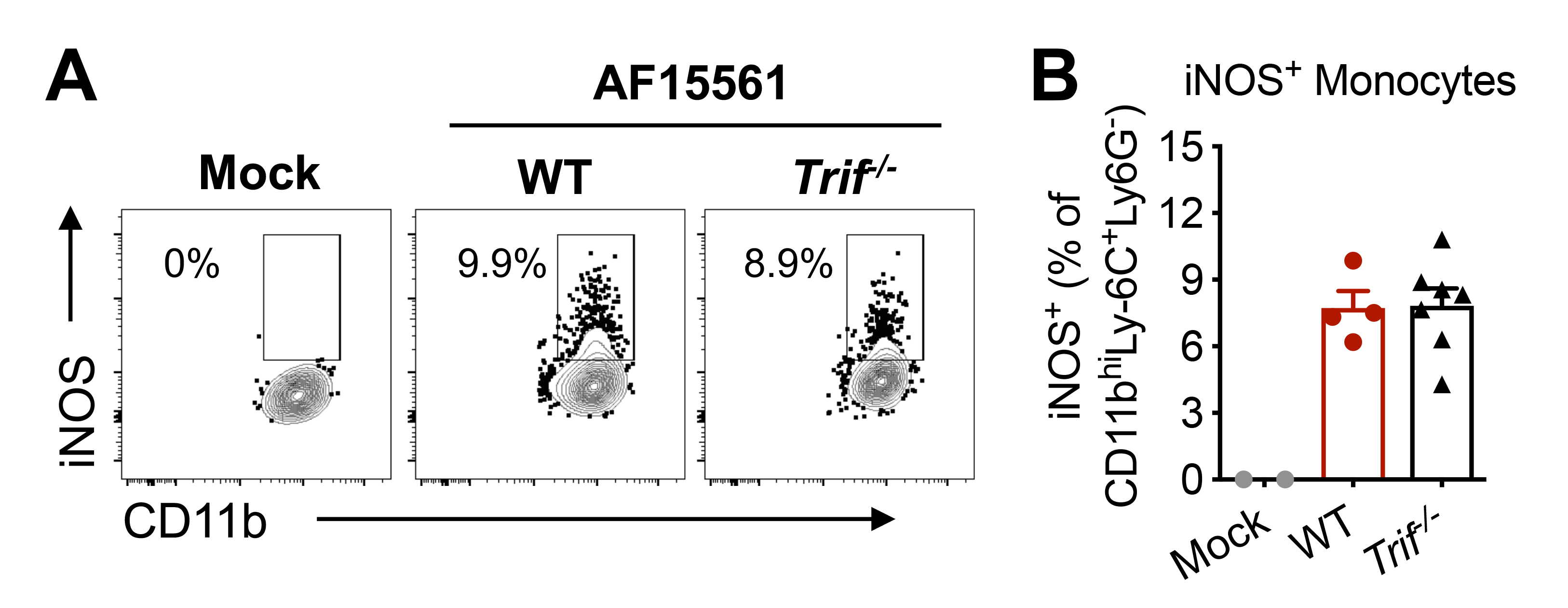

Supplement: S5 Fig — C57BL/6 mice were inoculated with PBS (mock) or with 103 PFU of CHIKV AF15561 in the left footpad and the dLN was analyzed at 24 hpi. (A) Percentage and (B) representative flow cytometry plots of CD11b+Ly6Chi monocytes expressing iNOS in WT or Trif-/- mice. Data are combined from 2 independent experiments. (TIF) [file ppat.1008292.s005.tif]
