# Supplementary material for: MyD88-dependent influx of monocytes and neutrophils impairs lymph node B cell responses to chikungunya virus infection via Irf5, Nos2 and Nox2
Source: PLoS Pathog. 2020 Jan 30;16(1):e1008292. doi: 10.1371/journal.ppat.1008292 (PMC7012455; doi:10.1371/journal.ppat.1008292)
Supplement: Attachment — Submitted filename: McCarthy et al-Response to Reviewer Comments-FINAL.pdf [file ppat.1008292.s006.pdf]

RESPONSE TO REVIEWER COMMENTS

REVIEWER 1

SUMMARY

In this study by McCarthy et al., the authors follow up on a published observation that infection with a pathogenic strain of CHIKV leads to an overall disorganization of the dLN including the failure of lymphocytes to accumulate and GCs to develop. Here, the authors perform a detailed, mechanistic analysis of what leads to this phenotype and show nicely that in mice infected with a "pathogenic" strain AF15561 there are increases in monocytes and neutrophils in the dLN early in infection and depletion of these cells leads to an accumulation of lymphocytes and restoration of the dLN organization after CHIKV infection. They go on to show that depletion of Gr-1 positive cells also increases CHIKV specific B-cell responses. To continue their mechanistic studies, they perform a beautiful set of genetic experiments to show that the monocyte recruitment is dependent on MyD88, Nos2 and Nox2 are involved in the disruption of the dLN during CHIKV infection, and iNos expression is controlled by an IFNAR and IRF5 dependent pathway. Finally, they show that there are distinct pathways activation ISGs in the footpad (IRF3/IRF7 dependent) and the dLN (IRF5 dependent). Overall, this study was well-written, easy to follow, and is novel, contributing to multiple areas of CHIKV infection. However, I had a few concerns that will should be addressed.

We appreciate the positive summary by the reviewer.

MAJOR COMMENTS

1. The authors make the point several times that the clearance of attenuated, acute CHIKV infections are due to B-cell and Ab responses (Lines 80-81 for example) and that during persistent, pathogenic CHIKV infections "virus-specific B-cell responses may be impaired" (Lines 82-83). I took this as an important point of the paper and that the authors were hypothesizing that the impaired B cell response is what is responsible or partly responsible for persistence of pathogenic CHIKV. The authors do address B-cell specific responses in Figure 4 and nicely show increases in CHIKV specific B-cells. However, it is unclear if this increase in B cells leads to increased clearance of pathogenic CHIKV and is a potential mechanism for persistence. The authors should measure viral titers in Gr-1 depleted mice to determine if pathogenic CHIKV has increased clearance after monocyte depletion and B cell recruitment. If this was not the point of the paper the authors may want to focus the writing on the disorganized dLN, etc phenotype and leave out viral clearance and persistence until the discussion. If this was addressed but didn't work, the authors should include some lines in the discussion about potential mechanisms.

We appreciate these reviewer concerns, and note that they have been addressed to some extent in prior publications. In previous studies, we found that the attenuated CHIKV 181/25 strain, in contrast to its parental pathogenic CHIKV strain AF15561, is cleared from WT mice but not from mice lacking the capacity to produce virus-specific antibody, suggesting that B cell responses are essential for clearance of 181/25 infection and that the pathogenic AF15561 strain evades these clearance mechanisms [1]. Moreover, the differential clearance of the two strains in WT mice was associated with differences in the quality of the CHIKV-specific antibody response [1]. For these reasons, we performed studies to evaluate the development of the immune response in the draining lymph node (dLN), the first lymphoid organ to encounter antigen following infection, after infection with either attenuated or pathogenic CHIKV strains.

*In our previous studies, we found that infection with pathogenic CHIKV strains disrupts dLN architecture and impairs accumulation of naïve lymphocytes [2]. In this study, we identify the cellular and molecular mechanisms that cause disruption of dLN architecture and lymphocyte accumulation during pathogenic CHIKV infection. In addition, we evaluated their impact on important aspects of the B cell response including the development of GCs, antibody-secreting cells, and plasma cells in the dLN. Overall, the experiments revealed that a MyD88-dependent influx of monocytes and neutrophils disrupts lymph node organization and impairs lymph node B cell responses via IRF5-dependent, IRF3/7-independent, activation of lymph node monocytes, iNOS, and NOX2. We stated in the Introduction section that "the mechanisms leading to dLN architectural disruption and impaired lymphocyte accumulation remain to be defined" (**lines 87-88**), indicating that this was the main goal of our*

*investigations. We have been careful with the conclusions made from the data outlined in this manuscript, noting only that the influx of myeloid cells to the dLN reduces the accumulation of lymphocytes, disrupts lymphocyte organization, and impairs the development of virus-specific B cell responses in the dLN (**lines 89-91, 291-294, and 391-393**). To temper our conclusions, we have changed the final sentence of the manuscript to reflect that our findings "underscore how local innate immune responses in draining lymphoid tissue dictate the <u>magnitude</u> of downstream adaptive immunity" (**lines 397-398**).*

*To further address the reviewer's concerns and to highlight the importance of a successful LN response for anti-CHIKV immunity, we infected congenic lymphotoxin alpha-deficient (LTα$^{-/-}$) mice, which lack peripheral LNs [3], with the attenuated CHIKV 181/25 strain (see **new Fig S1**). Whereas 181/25 is cleared or near the limit of detection in WT mice by 28 dpi, virus persists in the right ankle of LTα$^{-/-}$ mice at levels similar to that of WT mice infected with the pathogenic AF15561 strain. These data further emphasize the consequence of the failure to mount an efficient LN response for clearance of CHIKV from joint-associated tissues.*

We appreciate the reviewer's suggestion to measure viral titers in mice treated with anti-Gr1 antibody. However, the interpretation of these data is complicated by several factors. First, monocytes infiltrate joint-associated tissue during infection with CHIKV and other arthritogenic alphaviruses and have antiviral effects at this site through the production of type I IFN [4]. Additionally, monocytes are the effector cell required for monoclonal Ab-mediated clearance of CHIKV [5]. To address this question with a more targeted approach, we are in the process of generating Nox2$^{fl/fl}$ and Nos2$^{fl/fl}$ mice to specifically delete expression of these genes in monocytes and/or neutrophils, which should show reduced disruption of dLN B cell responses and allow us to assess the impact on viral loads without altering other important functions of these cells in the antiviral response. These studies are beyond the scope of this report.

2. In the title and their conclusions, the authors make the point that this MyD88-dependent recruitment of monocytes impairs CHIKV B-cell responses. However, the authors only show CHIKV specific B cells after Gr-1 treatment. What about in My88 deficient mice? Nos, Nox, and IRF5 deficient mice? The authors should address lymphocyte recruitment to dLN and CHIKV Abs in these mice.

*We addressed lymphocyte recruitment to the dLN in Nos2- and Nox2-deficient mice in **Figure 6A**, demonstrating that dLN lymphocyte numbers are increased in the absence of either Nos2 or Nox2. However, measurements beyond 5 dpi become complicated in whole-body knockout mice. For example, Nos2 expression by fibroblastic reticular cells in the LN can dampen expansion of T cells during acute inflammation [6], and B cell-intrinsic MyD88 expression enhances the antibody response to vaccination [7]. There are likely roles in multiple cell types for MyD88, Nos2, Nox2, and IRF5 on inflammation and viral replication either locally in joint-associated tissues or in the dLN as infection progresses that would obscure effects on CHIKV-specific Ab responses.*

As a more targeted approach to determine the specific effect of Nos2 and Nox2 expression in myeloid cells on CHIKV-specific Ab responses in the dLN, our laboratory is generating LysM^{Cre}xNox2$^{fl/fl}$ and LysM^{Cre}xNos2$^{fl/-}$ mice. Additionally, we are working to cross Irf5$^{fl/fl}$ mice with CD11c^{Cre} mice or other Cre-driver strains to determine the cell type requirement for IRF5 to induce Nos2 expression in infiltrating monocytes. Upon identification of the IRF5-expressing cell in the dLN, we can then use mice with Cre-driven deletion of IRF5 in that specific cell type to measure downstream CHIKV-specific Ab responses as opposed to a whole-body knockout approach.

3. The use of “attenuated” and “pathogenic, persistent” to describe the CHIKV strains was a bit confusing. There are other pathogenic strains of CHIKV that may not be persistent strains (IOL for example) and relative to each other, some strains may be considered “attenuated”. At this point, it is unclear if this same mechanism holds up for all of these strains and until it’s addressed I suggest calling these viruses as they are “vaccine” and “AF15561” or “parent”.

*To help clarify, we have now referred to the two CHIKV strains used in this manuscript as the “pathogenic (parental strain AF15561) or attenuated (derivative strain 181/25” (**see line 111**), and have applied the terms “pathogenic” and “attenuated” to AF15561 and 181/25, respectively for the remainder of the manuscript for the sake of simplicity. Our laboratory has shown that CHIKV strains from all lineages, including multiple IOL strains, are capable of establishing persistent infections in joint-associated tissue of mice (**Fig R1** and [8]). These data have been corroborated by multiple groups, which also have shown persistence of CHIKV strains from the ECSA-IOL lineage [9-11]. Relative to the pathogenic, parental AF15561 strain and to every other WT CHIKV strain, 181/25 is attenuated in immunocompetent mice.*

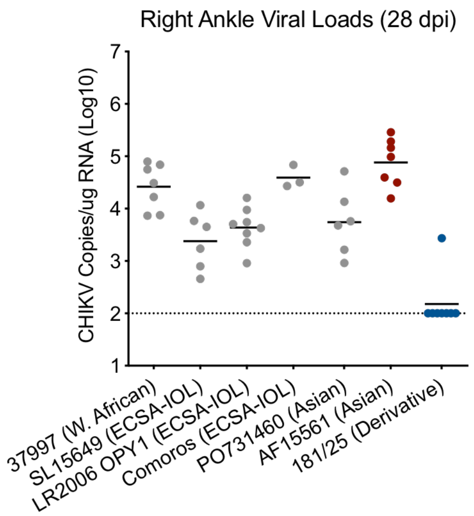

Fig R1. WT mice were infected with 10^3 PFU of the indicated CHIKV strain in the left footpad. At 28 days post-inoculation, viral loads in the right ankle were measured by RT-qPCR.

MINOR COMMENTS

1. Figure 1B: There is a * as significant that is not reflected in the figure legend.

*Thank you for bringing this to our attention. We have corrected this in the legend text (**see line 742**).*

2. Figure 7: The figure legend is swapped with the figure. For example, Figure 7F is described in what is B in the legend. Figure G-H are described in C-D of the legend.

*Thank you for pointing out this discrepancy. The legend text has been altered to reflect the correct figure order (**see lines 818-826**).*

REVIEWER 2

SUMMARY

Previously, this group has shown that pathogenic CHIKV strains persist in the joint tissues of WT mice while the attenuated vaccine strain was cleared in a fashion dependent on virus-specific antibody responses. In this study, McCarthy et. al. identified two cell types, neutrophils and inflammatory monocytes, that when depleted increase lymphocyte accumulation and improve draining lymph node (dLN) organization and CHIKV-specific B cell responses prior to infection with a pathogenic and chronic CHIKV strain (AF15561). This is due to iNOS and NOX2 expression in a IRF5/IFNAR1-dependent and partially TLR7-dependent mechanism.

This work is important and novel in two main ways. The authors have 1) defined the cell types and pathways responsible for differential B cell responses to acute versus chronic CHIKV infections, and 2) identified differential innate immune gene expression programs in the site of inoculation versus dLN. This study has left some interesting remaining questions: 1) what are the MyD88-dependent pathways in addition to TLR7, and 2) what is the cell type requirement for IRF5? The manuscript is well-written, and the model is nicely developed and supported by data that demonstrates both rigor and statistical power. Overall, this is a very nice study. Only a few issues need to be addressed.

We thank the reviewer for their positive comments.

MAJOR COMMENTS

1. It is not clear what the molecular mechanism and functional consequences of improved B cell responses are. In the previous study (Hawman et al, Cell Rep 2016), the pathogenic CHIKV strain AF15561 has evolved ways to evade neutralizing antibody responses against E2. How does myeloid cell depletion in AF15561-infected mice improve neutralization activity of serum? Has the virus mutated? Is this increase in serum neutralization activity sufficient to restrict viral replication in the joint tissues and facilitate viral clearance? It would be informative to perform passive transfer of serum from Gr-1 antibody treated mice and include more discussions on this data.

*These are all interesting questions. We have shown that myeloid cells inhibit CHIKV-specific neutralizing Ab responses by 1) impairing lymphocyte recruitment to the dLN (**Figure 2G-J**), and 2) disrupting dLN architecture (**Figure 2K**). In **Figure 4**, we showed that myeloid cell depletion resulted in improved GC formation and increased plasma cell numbers in the dLN, which likely are direct downstream consequences of the improved lymphocyte recruitment and dLN architecture earlier during infection. In **Figure S3**, we showed that myeloid cell depletion had modest effects on the GC B cell and plasma cell response in the inguinal LN and spleen. However, the most pronounced effects occurred in the dLN (**Figure 4**), suggesting that the modestly improved neutralizing activity of the serum observed following myeloid cell depletion is primarily due to the increased GC B cell and plasma cell response within the dLN itself. The data in **Figure 4M** were simply intended to show that differences in the dLN Ab response are measurable as a modest systemic improvement (**lines 181-182**), while the main focus of the paper was on responses in the dLN.*

*We appreciate the reviewer's suggestion to measure viral titers in mice treated with anti-Gr1 antibody. However, as outlined above in response to Reviewer 1 Comment 1, the interpretation of these data is complicated by several factors. First, monocytes infiltrate joint-associated tissue during infection with CHIKV and other arthritogenic alphaviruses and have antiviral effects at this site through the production of type I IFN [4]. Additionally, monocytes are the effector cell required for monoclonal Ab-mediated clearance of CHIKV [5]. To address this question with a more targeted approach, we are in the process of generating $Nox2^{fl/fl}$ and $Nos2^{fl/fl}$ mice to specifically delete expression of these genes in monocytes and/or neutrophils, which should minimize disruption of dLN B cell responses and allow us to assess the impact on viral loads without altering other important functions of these cells in the antiviral response. These studies are beyond the scope of this report. In the interim, as outlined above, we infected congenic lymphotoxin alpha-deficient ($LT\alpha^{-/-}$) mice, which lack peripheral LNs [3], with the attenuated CHIKV 181/25 strain (see **new Fig S1**). While 181/25 is cleared or nearly at the limit of detection in WT mice by 28 dpi, it persists in the right ankle of $LT\alpha^{-/-}$ mice at levels similar to that of WT mice infected with the pathogenic AF15561 strain. These data suggest that LN responses are essential for the clearance of CHIKV 181/25 infection.*

Finally, we agree with the reviewer that passive transfer of sera from control antibody and anti-Gr1 antibody treated mice could be informative. However, these experiments will require large amounts of sera and high numbers of mice in order to perform extensive titration analyses and are not feasible to include as a component of this already extensive report focused on the cellular and molecular mechanisms driving disruption of the dLN during CHIKV infection.

2. In Figure 1A, dLN from AF15561-infected mice has significantly more cells in general. What are these cells other than the neutrophils and monocytes? It would be informative to immunophenotype all the cells in the dLN. Since depletion of both neutrophil and monocytes does not completely rescue CD8+ T cells from the dLN (Fig. 2K) and IRF5 is required and constitutively expressed in DCs, it is possible that DCs might play a role in viral persistence. Does pathogenic CHIKV infection increase the influx of DCs into dLN?

*The dLN of CHIKV AF15561-infected mice likely has more total cells at 24 hpi due to type I IFN-induced inhibition of lymphocyte egress, as the dLN of IFNAR1-deficient mice has fewer total cells than WT mice (**Figure 5A**). The increased number of total cells following CHIKV AF15561 infection reflects in an increase in the number of nearly all cell types examined, including B and T cells, without a corresponding increase in the percentage of total. This is consistent with higher levels of type I IFN in the dLN of CHIKV AF15561-infected mice compared with 181/25-infected mice (unpublished results).*

DCs do increase in number in the dLN, although whether this is due to specific recruitment of these cells or prevention of their egress is not known. DCs may play a role in the phenotypes observed. IRF5 is highly expressed in classical DCs (as the reviewer noted), pDCs, and B cells, although it can be induced in other cell types, including myeloid cells [12-17]. We are working to cross Irf5$^{fl/fl}$ mice with CD11c^{Cre} mice or other Cre driver strains to determine the cell type requirement for IRF5 in ISG induction, monocyte activation, and the downstream phenotypes of LN disorganization and impaired dLN B cell responses.

MINOR COMMENTS

1. In a few occasions, the data does not fully support the model and the writing needs to be modified to take that into consideration. For example, in lines 209-210, the authors stated that "IL-1R-independent, MyD88-dependent signal(s) promote the recruitment of monocytes to the dLN". However, monocyte number is significantly reduced in the dLN of IFNAR knockout mice although the percentage of monocytes is not affected, suggesting that IFN signaling might have an effect on monocyte accumulation. Additionally, most of the ISGs tested in Figure 8 demonstrate strict IRF5 dependency in dLN except for Ifit1 and Rsad2. Is it possible that the dichotomy of IRF3/7-dependent ISG expression near site of inoculation and IRF5-dependent ISG expression in dLN is gene specific?

*Ifnar1$^{-/-}$ mice are unable to undergo type I IFN-induced inhibition of lymphocyte egress [18] (**lines 191-193**), a mechanism to prevent egress of circulating immune cells at the start of an immune response, and thus have fewer total cells in the dLN than WT mice. Because the percentage of monocytes is not affected in IFNAR1-deficient mice, the reduction in monocyte number is due to the reduction in total dLN cells from the lack of this general shutdown mechanism rather than a specific effect of type I IFNs on monocyte accumulation.*

*Studies in virally infected IRF5 or IRF7 overexpressing cells have shown that IRF5 activates distinct IFN-α genes compared to IRF7 [17]. In addition, a subsequent study identified a large number of common genes activated by these two IRFs in response to viral infection [19]. However, there also were several distinct, non-overlapping genes induced by IRF5 versus IRF7, or genes that were induced more effectively by IRF5 than IRF7 (one of which was Rsad2). Thus, the difference in IRF3/7 versus IRF5 dependency may be somewhat gene-specific, in that induction of Ifit1 and Rsad2 are only partially IRF5-dependent in the dLN. This difference between Ifit1 and Rsad2 and the other genes analyzed has been noted in the text (**lines 283-284**).*

2. Lines 27-28 should be changed to "Infection of WT mice with pathogenic but not acutely cleared CHIKV induced MyD88-dependent recruitment".

We have changed as requested by the reviewer.

3. In lines 189-190, the use of double negative is confusing and difficult to understand.

We have modified this text for clarity.

4. Line 237 should refer to Fig 6G.

Thank you. This has been corrected in the text.

REVIEWER 3

SUMMARY

The report from McCarthy et al describes mechanisms behind differences in the control of pathogenic versus attenuated chikungunya virus strains. Multiple transgenic mouse strains are used in this detailed description of the events up- and downstream of the role monocytes and neutrophils play in disrupting effective B-cell responses in draining lymph nodes. This is an excellent, and very thorough study, likely to be of general interest and highly impactful for the field.

We thank the reviewer for their positive summary.

The differences between attenuated and pathogenic virus in terms of monocyte recruitment into draining LNs, the mechanisms behind this and the downstream consequences to LNs and germinal center formation are all clearly described and supported. The only criticism of the study is that although there are claims that these local innate immune responses "…..dictate the effectiveness of downstream adaptive immunity", there is very little actual evidence to support this. There is no discussion as to whether and how these minimal changes may represent the stark differences in pathogenicity seen. More detailed time-courses, isotype differences and/or study of anti-CHIKV memory B-cells or long-lived plasma cells from outside the LN may also identify more significant differences. There is also no discussion of impact on disease outcomes in the mice. Obviously there are issues with downstream effects on viral clearance by these cells in tissues, however with careful timing, or in some of the mouse strains, it seems like some mitigation of disease should be observed if the germinal center disruption really plays a significant role in pathogenesis. Despite this caveat, the study remains highly informative and will move the field forward significantly.

We refer the reader to our response to ***Reviewer 1, Major Comment 1****.*

MAJOR COMMENTS

None.

MINOR COMMENTS

Reviewer #3: Fig. 4L and M would be more informative with absolute levels demonstrated, rather than fold change which can be deceiving.

Due to differences in the absolute $FRNT_{50}$ values between experiments, fold change has been used in order to combine data from multiple experiments. This was not meant to be deceiving. Rather, we feel that fold change as currently displayed is more intuitive for a reader to understand – that changes in the local dLN Ab response can be measured systemically as a modest 2-fold qualitative improvement.

LITERATURE CITED

1. Hawman DW, Fox JM, Ashbrook AW, May NA, Schroeder KMS, Torres RM, et al. Pathogenic Chikungunya Virus Evades B Cell Responses to Establish Persistence. Cell Rep. 2016;16(5):1326-38. Epub 2016/07/28. doi: 10.1016/j.celrep.2016.06.076. PubMed PMID: 27452455; PubMed Central PMCID: PMCPMC5003573.
2. McCarthy MK, Davenport BJ, Reynoso GV, Lucas ED, May NA, Elmore SA, et al. Chikungunya virus impairs draining lymph node function by inhibiting HEV-mediated lymphocyte recruitment. JCI Insight. 2018;3(13). Epub 2018/07/13. doi: 10.1172/jci.insight.121100. PubMed PMID: 29997290; PubMed Central PMCID: PMCPMC6124534.
3. De Togni P, Goellner J, Ruddle NH, Streeter PR, Fick A, Mariathasan S, et al. Abnormal Development of Peripheral Lymphoid Organs in Mice Deficient in Lymphotoxin. Science. 264(5159 (Apr. 29, 1994)):703-7.
4. Haist KC, Burrack KS, Davenport BJ, Morrison TE. Inflammatory monocytes mediate control of acute alphavirus infection in mice. PLoS Pathog. 2017;13(12):e1006748. Epub 2017/12/16. doi: 10.1371/journal.ppat.1006748. PubMed PMID: 29244871; PubMed Central PMCID: PMCPMC5747464.
5. Fox JM, Roy V, Gunn BM, Huang L, Edeling MA, Mack M, et al. Optimal therapeutic activity of monoclonal antibodies against chikungunya virus requires Fc-FcgammaR interaction on monocytes. Science Immunology. 2019;4(eaav5062 (2019)).
6. Siegert S, Huang HY, Yang CY, Scarpellino L, Carrie L, Essex S, et al. Fibroblastic reticular cells from lymph nodes attenuate T cell expansion by producing nitric oxide. PLoS One. 2011;6(11):e27618. Epub 2011/11/24. doi: 10.1371/journal.pone.0027618. PubMed PMID: 22110693; PubMed Central PMCID: PMCPMC3215737.
7. AL D, DC R, B H. Contribution of Toll-like receptor signaling to germinal center antibody responses. Immunological Reviews. 2012;247:64-72.
8. Hawman DW, Stoermer KA, Montgomery SA, Pal P, Oko L, Diamond MS, et al. Chronic joint disease caused by persistent Chikungunya virus infection is controlled by the adaptive immune response. J Virol.

2013;87(24):13878-88. Epub 2013/10/18. doi: 10.1128/JVI.02666-13. PubMed PMID: 24131709; PubMed Central PMCID: PMCPMC3838294.
9. Pal P, Fox JM, Hawman DW, Huang YJ, Messaoudi I, Kreklywich C, et al. Chikungunya viruses that escape monoclonal antibody therapy are clinically attenuated, stable, and not purified in mosquitoes. J Virol. 2014;88(15):8213-26. Epub 2014/05/16. doi: 10.1128/JVI.01032-14. PubMed PMID: 24829346; PubMed Central PMCID: PMCPMC4135940.
10. Hong JP, McCarthy MK, Davenport BJ, Morrison TE, Diamond MS. Clearance of Chikungunya Virus Infection in Lymphoid Tissues Is Promoted by Treatment with an Agonistic Anti-CD137 Antibody. Journal of Virology. 2019;93(24). doi: 10.1128/JVI.01231-19CE.
11. Uhrlaub JL, Pulko V, DeFilippis VR, Broeckel R, Streblow DN, Coleman GD, et al. Dysregulated TGF-beta Production Underlies the Age-Related Vulnerability to Chikungunya Virus. PLoS Pathog. 2016;12(10):e1005891. Epub 2016/10/14. doi: 10.1371/journal.ppat.1005891. PubMed PMID: 27736984.
12. Takaoka A, Yanai H, Kondo S, Duncan G, Negishi H, Mizutani T, et al. Integral role of IRF-5 in the gene induction programme activated by Toll-like receptors. Nature. 2005;434(7030):243-9. Epub 2005/02/23.
13. Duffau P, Menn-Josephy H, Cuda CM, Dominguez S, Aprahamian TR, Watkins AA, et al. Promotion of Inflammatory Arthritis by Interferon Regulatory Factor 5 in a Mouse Model. Arthritis Rheumatol. 2015;67(12):3146-57. Epub 2015/09/01. doi: 10.1002/art.39321. PubMed PMID: 26315890; PubMed Central PMCID: PMCPMC4661118.
14. Weiss M, Byrne AJ, Blazek K, Saliba DG, Pease JE, Perocheau D, et al. IRF5 controls both acute and chronic inflammation. Proc Natl Acad Sci U S A. 2015;112(35):11001-6. Epub 2015/08/19. doi: 10.1073/pnas.1506254112. PubMed PMID: 26283380; PubMed Central PMCID: PMCPMC4568217.
15. Coccia EM, Severa M, Giacomini E, Monneron D, Remoli ME, Julkunen I, et al. Viral infection and Toll-like receptor agonists induce a differential expression of type I and lambda interferons in human plasmacytoid and monocyte-derived dendritic cells. Eur J Immunol. 2004;34(3):796-805. Epub 2004/03/03. doi: 10.1002/eji.200324610. PubMed PMID: 14991609.
16. Chow KT, Wilkins C, Narita M, Green R, Knoll M, Loo YM, et al. Differential and Overlapping Immune Programs Regulated by IRF3 and IRF5 in Plasmacytoid Dendritic Cells. J Immunol. 2018;201(10):3036-50. Epub 2018/10/10. doi: 10.4049/jimmunol.1800221. PubMed PMID: 30297339; PubMed Central PMCID: PMCPMC6219909.
17. Barnes BJ, Moore PA, Pitha PM. Virus-specific activation of a novel interferon regulatory factor, IRF-5, results in the induction of distinct interferon alpha genes. J Biol Chem. 2001;276(26):23382-90. Epub 2001/04/17. doi: 10.1074/jbc.M101216200. PubMed PMID: 11303025.
18. Gresser I, Guy-Grand D, Maury C, Maunoury M. Interferon induces peripheral lymphadenopathy in mice. J Immunol. 1981;127:1569-75.
19. Barnes BJ, Richards J, Mancl M, Hanash S, Beretta L, Pitha PM. Global and distinct targets of IRF-5 and IRF-7 during innate response to viral infection. J Biol Chem. 2004;279(43):45194-207. Epub 2004/08/17. doi: 10.1074/jbc.M400726200. PubMed PMID: 15308637.
